# Cost-effectiveness in diagnosis of stable angina patients: a decision-analytical modelling approach

**DOI:** 10.1136/openhrt-2021-001700

**Published:** 2022-04-04

**Authors:** Muhummad Sohaib Nazir, Yael Rodriguez-Guadarrama, Tiago Rua, Khan Ha Bui, Anna Buylova Gola, Amedeo Chiribiri, Paul McCrone, Sven Plein, Mark Pennington

**Affiliations:** 1 Biomedical Engineering and Imaging Sciences, King's College London, London, UK; 2 Centre for Medical Engineering, KiTEC – King’s Technology Evaluation Centre, King's College London, London, UK; 3 Centre for Medical Engineering, School of Biomedical Engineering and Imaging Sciences, King's College London, London, UK; 4 Institute for Lifecourse Development, Faculty of Education, Health & Human Sciences, University of Greenwich, London, UK; 5 Leeds Institute of Cardiovascular and Metabolic Medicine, University of Leeds, Leeds, UK; 6 King’s Health Economics, Institute of Psychiatry, Psychology & Neuroscience, King’s College London, London, UK

**Keywords:** CORONARY ARTERY DISEASE, STABLE ANGINA, Health Care Economics and Organizations

## Abstract

**Objective:**

Given recent data on published diagnostic accuracies, this study sought to determine the most cost-effective diagnostic strategy for detection of significant coronary artery disease (CAD) in stable angina patients using invasive coronary angiography (ICA) and fractional flow reserve (FFR) as the reference standard.

**Methods:**

A probabilistic decision-analytical model was developed which modelled a cohort of patients with stable angina. We investigated 17 diagnostic strategies between standalone and combination of different imaging tests to establish a correct diagnosis of CAD, using no testing as the baseline reference. These tests included CT coronary angiography (CTCA), stress echocardiography, CT-based FFR, single-photon emission computed tomography (SPECT), cardiovascular magnetic resonance (CMR), positron emission tomography, ICA, and ICA with FFR. Incremental cost-effectiveness ratios were calculated as the additional cost per correct diagnosis.

**Results:**

SPECT followed by CTCA and ICA-FFR is the most cost-effective strategy between a cost-effectiveness threshold (CET) value of £1000–£3000 per correct diagnosis. CMR followed by CTCA and ICA-FFR is cost-effective within a CET range of £3000–£17 000 per correct diagnosis. CMR and ICA-FFR is cost-effective within a CET range of £17 000–£24 000. ICA-FFR as first line is the most-cost effective if the CET value exceeds the £24 000 per correct diagnosis. Sensitivity analysis showed that direct ICA-FFR may be cost-effective in patients with a high pre-test probability of CAD.

**Conclusion:**

First-line testing with functional imaging is cost-effective at low to intermediate value of correct diagnosis in patients with low to intermediate risk of CAD. ICA is not cost effective although ICA-FFR may be at higher CET.

**Key questions**

**What is already known about this subject?**

- Coronary artery disease (CAD) remains an important cause of morbidity and mortality.
- There is recent evidence on diagnostic accuracies of non-invasive imaging tests compared with invasive coronary angiography (ICA) with fractional flow reserve (FFR), the reference standard for significant CAD.
- We sought to determine the cost-effectiveness of different diagnostic strategies to guide clinical pathways over a range of pretest probabilities and cost-effectiveness thresholds.

**What does this study add?**

- Direct ICA alone for CAD testing is not cost effective, although direct ICA-FFR may be cost effective at high pretest probabilities.
- Non-invasive functional imaging with cardiovascular magnetic resonance is a cost-effective strategy for diagnosis of CAD, at low to intermediate pretest probability, assuming the willingness to pay for a correct diagnosis falls between £3000 and £24 000, and could be considered for first line testing for CAD.

**How might this impact on clinical practice?**

- These findings can help guide clinical decision making through the appropriate use of different diagnostic strategies according to patients’ pretest probabilities and the extent of healthcare funding.

## Introduction

Coronary artery disease (CAD) is the single most common cause of death worldwide^1^ and accurate and timely diagnosis of patients with suspected CAD is important to initiate optimal medical therapy and consider revascularisation, which may reduce adverse clinical outcomes. Several diagnostic tests are in clinical use for the detection of CAD. The current UK National Institute for Health and Care Excellence (NICE) guidelines propose CT coronary angiography (CTCA) as first line for investigation for chest pain of suspected cardiac origin.^2^ A more recently published guideline from the European Society of Cardiology (ESC) recommends functional or anatomical non-invasive imaging for detection of stable CAD with consideration of pretest probability.^3^ While the guidelines produced by NICE include recommendations based on economic analysis, the ESC guideline does not consider cost-effectiveness as part of the recommendations. An important limitation of the NICE guidelines is that the diagnostic accuracies presented as part of the health economic evaluation used invasive coronary angiography (ICA) as the reference standard, which determines anatomical information only rather than functional significance of coronary artery lesions. The reference test for detection of functionally significant CAD is invasive coronary angiography with fractional flow reserve (ICA-FFR). Management of patients with FFR-guided care is associated with better clinical outcomes compared with patients managed based on ICA alone.^4 5^ Based on contemporary meta-analyses of the diagnostic accuracy of anatomical and functional tests,^6 7^ we sought to determine the most cost-effective diagnostic strategy for the detection of functionally significant CAD in patients presenting with new onset stable angina using ICA-FFR as the reference standard.

## Methods

### Overview and model structure

**Figure 1:**
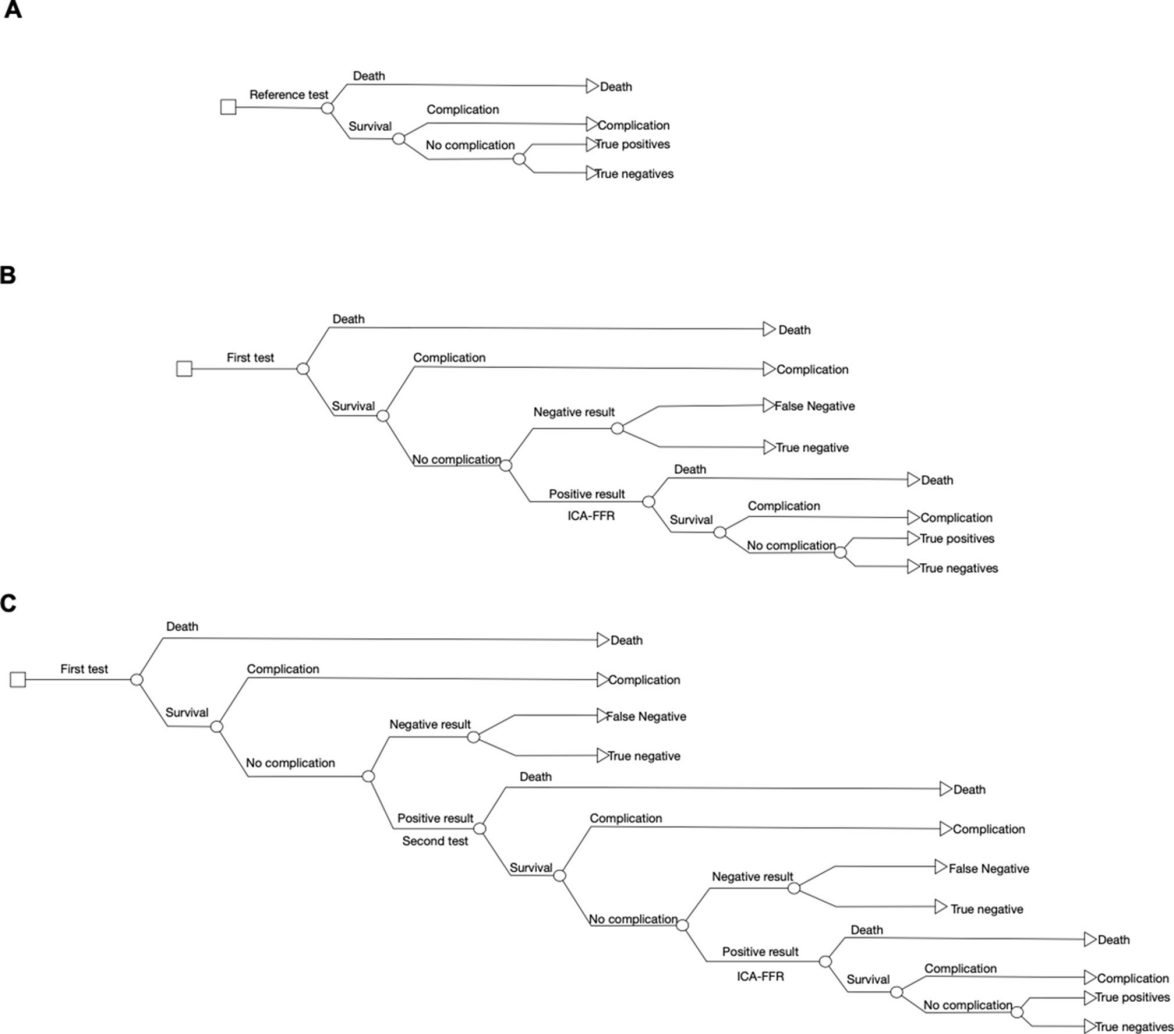
Model structure. (A) Reference test (ICA-FFR). (B) Combination of one test and reference test. (C) Combination of two tests and reference test. ICA-FFR, invasive coronary angiography with fractional flow reserve.

We developed a decision-analytical model to assess the cost-effectiveness of different diagnostic strategies in patients with suspected stable, functional CAD. A decision tree was built to capture the results of testing as the number of true and false positive and negative results, and the incidence of complications and death. The model considered a hypothetical cohort of patients with suspected stable CAD that undergo a diagnostic test. The model structure for the alternative combinations tested is reported in figure 1. The model considered a population with a prior risk of 37.6%, as the base case, based on a previous large study of patients that underwent coronary angiography for suspected chest pain.^8^ This risk is adjusted as part of the scenario analysis, described later. A healthcare perspective was applied. The time horizon extended to the initial sequence of tests and the treatment of complications arising from those tests. Given the short-term horizon, no discounting was undertaken.

The primary outcome was the expected proportion of correct diagnoses, a composite parameter calculated as the sum of true positive and true negative results. Patients who experienced either death or a complication were considered not to have received a correct diagnosis regardless of the test result. This approach implicitly considers death, a complication, a false negative or a false positive result to be equally detrimental outcomes.

### Testing strategies

We compared 17 diagnostic strategies based on (1) no testing, (2) anatomical followed by functional testing and then ICA-FFR (3) functional following by anatomical testing, and then ICA-FFR (online supplemental table A1). We considered ICA-FFR to be the reference test. The diagnostic tests included CTCA, stress echocardiography (SE), CT-based FFR, single-photon emission CT (SPECT), cardiovascular magnetic resonance (CMR), positron emission tomography, ICA and ICA with FFR (ICA-FFR). Negative results did not require further investigation. For clarity, from hereafter we refer to strategies as their test sequence (eg, CTCA+CMR+ICA-FFR).

10.1136/openhrt-2021-001700.supp1 Supplementary data

### Model parameters

The diagnostic accuracies of the different tests were obtained, from recent meta-analyses that used ICA-FFR as the reference standard,^6 7^ of which the largest meta-analysis was used to inform the recent ESC guideline on chronic coronary syndrome.^6 9^ The parameters that populated the model are shown in online supplemental table A2. The predictive values of each test were determined using the prevalence of the disease and the corresponding values of diagnostic accuracy. The probabilities of death and non-fatal complications were obtained from published evidence including large multicentre registries.^10–14^ All probabilities were considered independent, and the results of any previous testing did not influence the result of the subsequent testing. Furthermore, patients were assumed to be eligible for all diagnostic tests.

### Costs

Cost of each diagnostic test was taken from National Health Service (NHS) reference costs 2017/2018.^15^ The costs of complications were calculated as the weighted average of relevant Health Resource Group codes. Weighting was based on the frequency of each complication. Online supplemental table A2 summarises the costs used in the model. It was assumed that a fatal outcome of a test did not incur an additional cost.

### Cost-effectiveness analysis

We report cost-effectiveness as the cost per correct diagnosis. Using the strategy of ‘no testing’ as the baseline reference comparator, we calculated and presented incremental cost-effectiveness ratios (ICERs) for non-dominated diagnostic strategies for each subgroup. The ICER represents the additional cost per additional patient correctly diagnosed for a strategy compared with the next most effective strategy. Dominated and extendedly dominated strategies were eliminated prior to calculating of ICERs.^16^ Dominated strategies are those which generate poorer outcomes at a higher cost compared with an alternative strategy. Extendedly dominated strategies are those which generate poorer outcomes at higher cost compared with the application of an alternative strategy to some proportion of the population and a different alternative to the remaining population.

### Sensitivity analysis

Parametric uncertainty was captured with a probabilistic sensitivity analysis using 1000-iteration Monte Carlo simulation. In this approach, appropriate distributions were assigned to each parameter to reflect the underlying uncertainty in that parameter (online supplemental table A2), for example, with the confidence intervals for the diagnostic accuracies, costs of tests and complications costs. A value was then sampled from the specified distribution for each parameter and the model evaluated for that specific parameter set in each Monte Carlo simulation. The results of the simulation are reported using cost-effectiveness planes and cost-effectiveness acceptability curves (CEAC).^17^ The latter report the likelihood that each strategy is the most cost-effective strategy according to the value placed on the outcome - a correct diagnosis. ICERs were calculated after determining mean costs and mean outcomes for each strategy from the probabilistic analysis.

In scenario analysis, we considered patient subgroups with pre-test probability of CAD between 15% and 85% with 10% increments according to recent ESC guidelines. We defined optimal diagnostic strategies as those yielding the maximum net monetary benefit (most cost-effective) at CET values ranging from £2000 to £50 000 per correct diagnosis. We report the best strategy across the range of pre-test probabilities at each CET along with the expected costs, proportion of correct diagnoses and incidence of complication and death.

## Results

**Figure 2:**
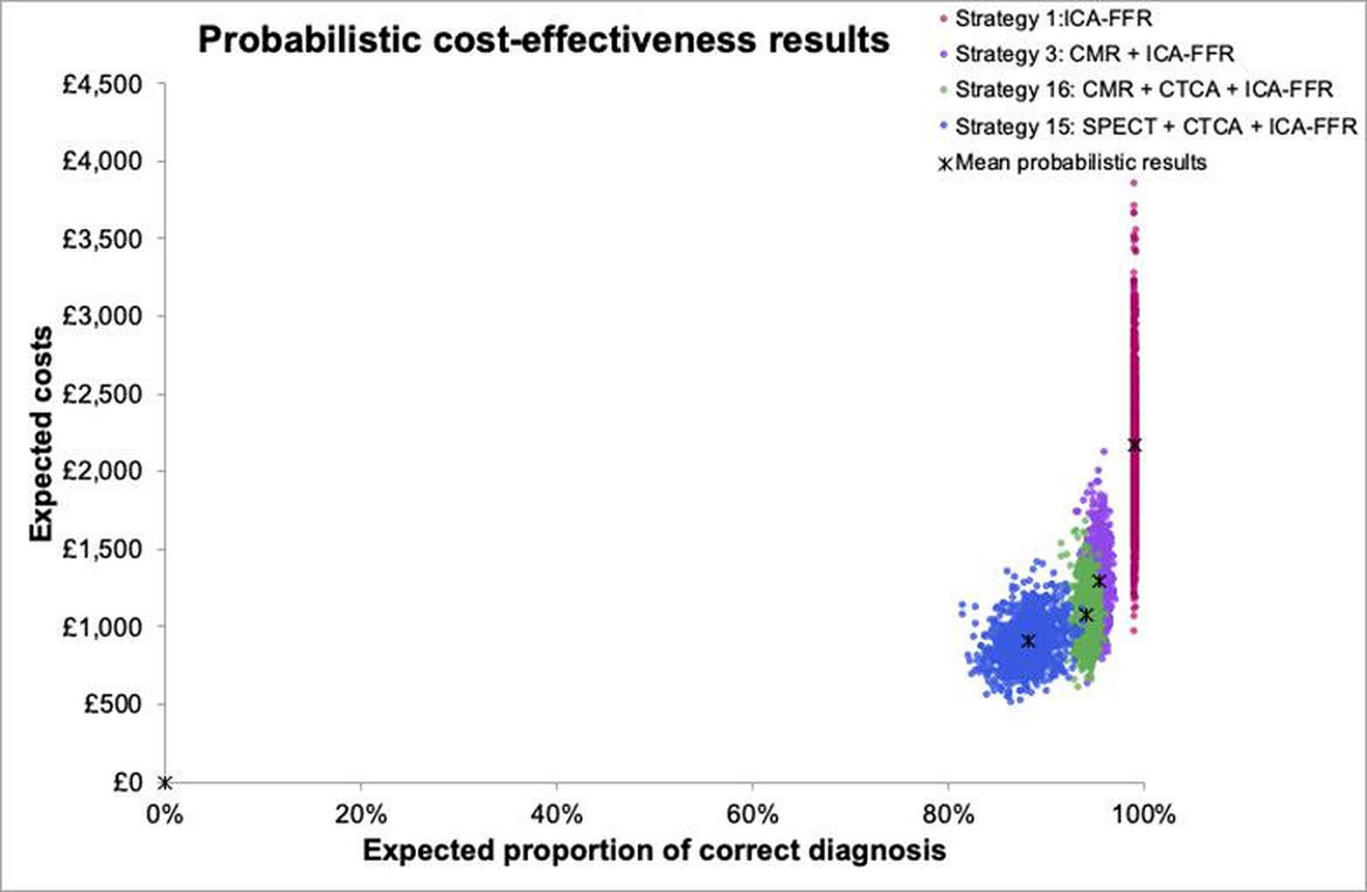
Probabilistic cost-effectiveness results. The solid line across panels represent the cost-effectiveness frontier. Strategy 1: ICA-FFR. Strategy 3: CMR+ICA FFR. Strategy 16: CMR+CTCA + ICA-FFR. Strategy 15: SPECT+CTCA + ICA-FFR. CMR, cardiovascular magnetic resonance; CTCA, CT coronary angiography; ICA-FFR, invasive coronary angiography and fractional flow reserve; SPECT, single-photon emission CT.

Costs, outcomes and cost-effectiveness of the non-dominated strategies in the base case are shown in table 1 and illustrated in figure 2. Results for all strategies, including dominated or extendedly dominated strategies, are shown in online supplemental table A3. Thirteen of the 17 strategies were dominated or extendedly dominated, suggesting they are not cost-effective.

**Table 1:** Probabilistic results of base-case scenario

Strategy no	Description	Expected deaths	Expected complications	Expected proportion of incorrect diagnoses	Expected proportion of correct diagnoses	Expected cost	Incremental cost per correct diagnosis	Probability of being the most cost effective strategy
1	ICA-FFR	0.08%	0.94%	0.00%	98.97%	£2176.02	£24 425.97	22%
3	CMR +ICA-FFR	0.03%	0.46%	4.16%	95.35%	£1291.80	£16 997.64	31%
16	CMR +CTCA + ICA-FFR	0.02%	0.65%	5.25%	94.08%	£1075.93	£2881.86	15%
15	SPECT+CTCA + ICA-FFR	0.02%	0.63%	11.16%	88.18%	£905.90	£1027.33	0.00%

CMR, cardiovascular magnetic resonance; CTCA, CT coronary angiography; ICA, invasive coronary angiography; ICA-FFR, invasive coronary angiography and fractional flow reserve; SPECT, single-photon emission CT.

From table 1, at a CET for a correct diagnosis of £1027 to £2,882, the most cost-effective strategy is SPECT+CTCA+ICA-FFR. If the CET for a correct diagnosis falls between £2882 and £16 998, the most cost-effective strategy was CMR+CTCA+ICA-FFR. Between £16 998 and £24 426, the strategy of CMR+ICA-FFR is the most cost-effective. First-line ICA-FFR yields the most correct diagnoses but is only cost-effective if the CET for a correct diagnosis exceeds £24 426.

### Impact of parameter uncertainty

**Figure 3:**
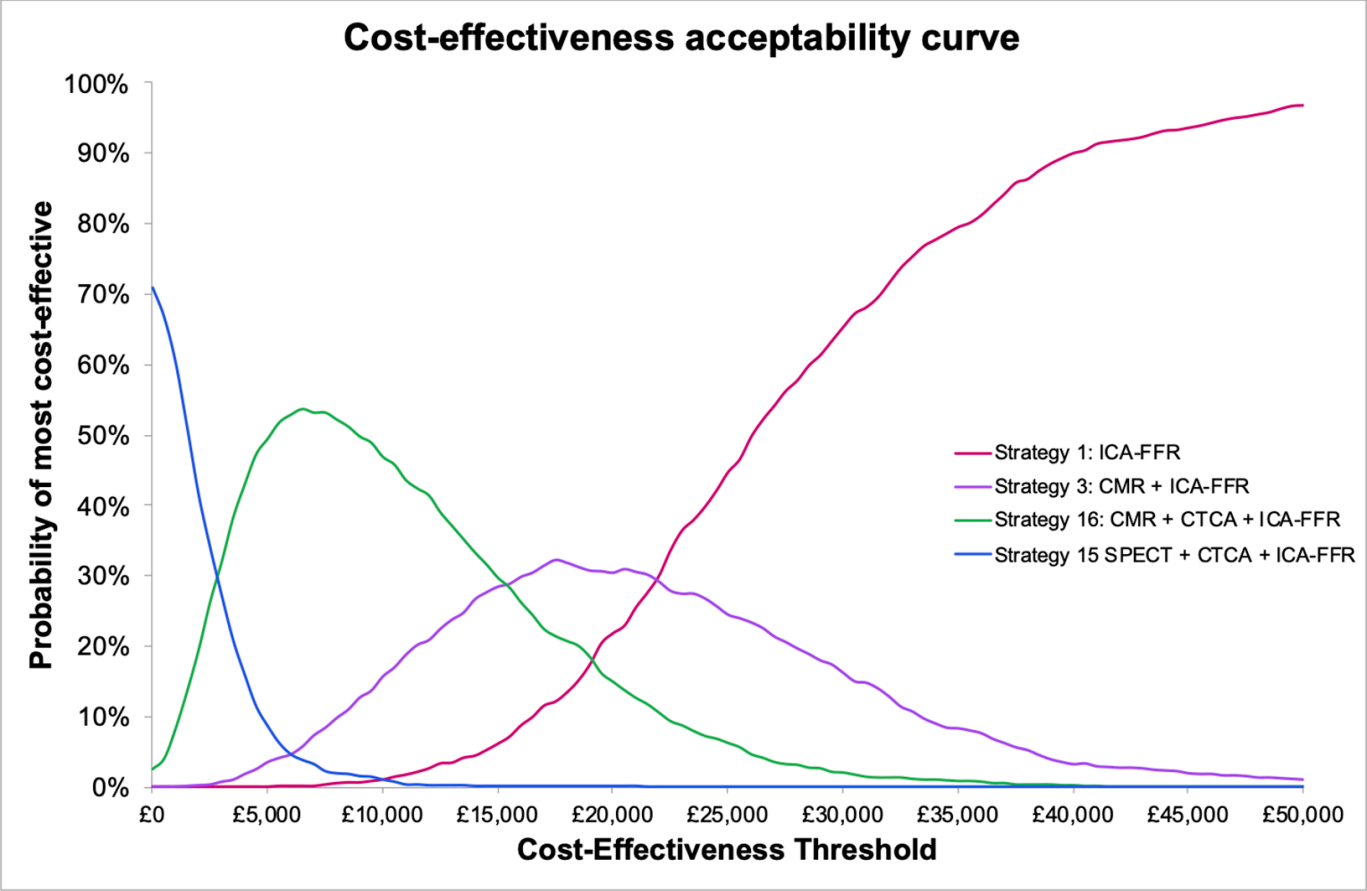
Cost-effectiveness acceptability curve. Strategy 1: ICA-FFR. Strategy 3: CMR+ICA FFR. Strategy 16: CMR+CTCA + ICA-FFR. Strategy 15: SPECT+CTCA + ICA-FFR. CMR, cardiovascular magnetic resonance; CTCA, CT coronary angiography; ICA-FFR, invasive coronary angiography and fractional flow reserve; SPECT: single-photon emission CT.

Figure 3 shows the CEAC for the base case analysis. For clarity, we plotted only the non-dominated strategies. The plot indicates considerable uncertainty in the likelihood that any one strategy is the most cost-effective across the mid range of CET values. At very low CET values for a correct diagnosis there is a high likelihood that SPECT+CTCA+ICA-FFR is the most cost-effective strategy. The likelihood of CMR+CTCA+ICA-FFR being the optimal strategy is high between £3000 and £15 000 per correct diagnosis. Over the range of £15 000–£22 000 per correct diagnosis, testing with CMR+ICA FFR is the most likely to be cost-effective. However, there is considerable uncertainty over this range, reflecting the number of reasonable alternative testing strategies compared. At values above £22 000 per correct diagnosis, the probability that ICA-FFR alone is the most cost-effective strategy rises rapidly.

### Scenario analysis according to pretest probability of CAD

**Figure 4:**
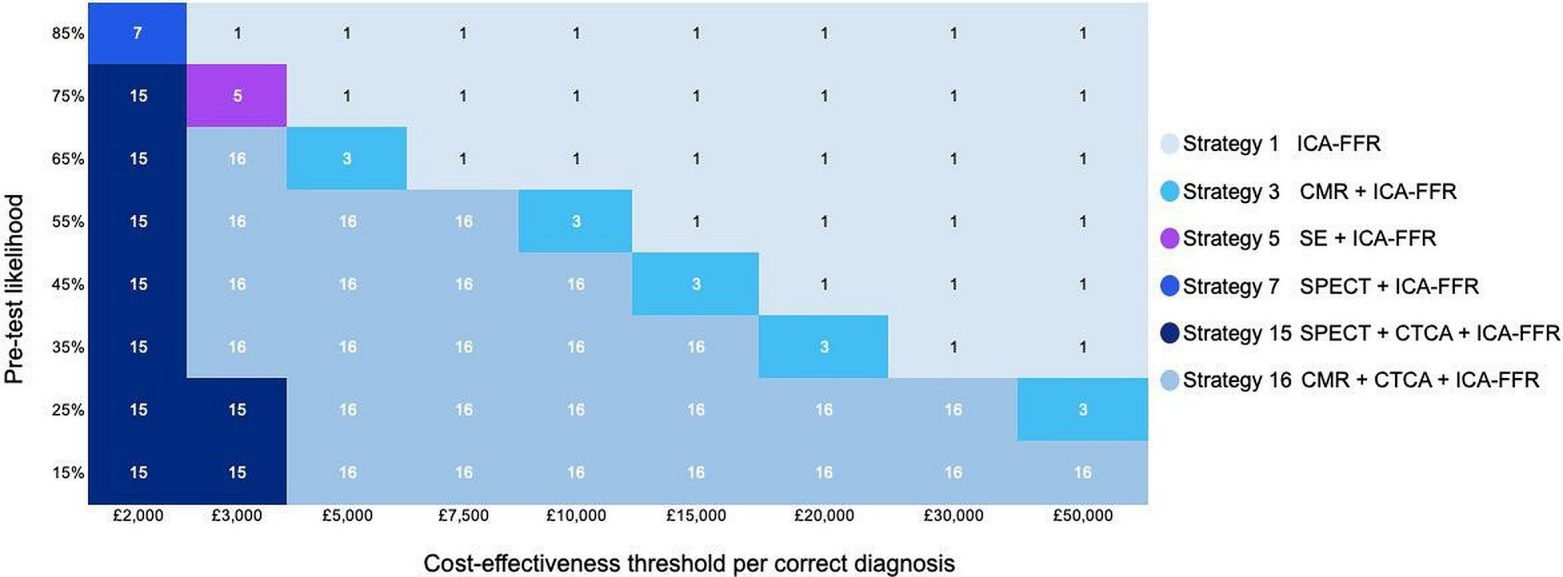
Scenario analysis on pretest likelihood and cost-effectiveness threshold per correct diagnosis.

Figure 4 and online supplemental table A4 present the results of scenario analysis examining a range of pretest probabilities of CAD. At a low CET value of £2000 per correct diagnosis, the strategy SPECT+CTCA+ICA-FFR is cost-effective across the range of pretest risk up to 85%. As the CET increases, CMR+CTCA+ICA-FFR is cost-effective over a growing range of pretest probabilities and rapidly replaces SPECT+CTCA+ICA-FFR as the cost-effective strategy even for low pretest probabilities. ICA-FFR is cost-effective at a high pre-test risk of 85% at a CET as low as £3000. As the CET increases, ICA-FFR becomes cost-effective at lower pre-test probabilities, gradually displacing CMR+CTCA+ICA-FFR. The ranking of cost-effective strategy remains broadly the same as pre-test probability of disease is varied. ICERs fall as pretest risk increases, increasing the likelihood that more expensive strategies such as ICA-FFR will be cost-effective.

## Discussion

Our analysis shows that first line testing with CMR is cost-effective if the additional cost per diagnosis falls in the range of £3000 to £24 000. These results reflect low to intermediate pretest probability of CAD. However, our sensitivity analysis demonstrates that at very high pretest probability of CAD, direct ICA-FFR may be cost-effective.

This study provides important insights for clinical decision-makers for the diagnosis of functional CAD from a cost-effectiveness perspective: (1) non-invasive functional testing is generally more cost-effective than invasive testing and direct ICA is not a cost-effective strategy; (2) SPECT and CMR are cost-effective first-line options at low CET values per correct diagnosis and within low risk subgroups, respectively and (3) direct ICA-FFR is cost-effective if a correct diagnosis is valued above £24 000.

Our findings challenge the recommendations presented in the most recent NICE guidelines on chest pain, which recommends CTCA as first line.^2^ The results suggest a greater role for non-invasive functional testing. This may in part be explained by a key difference between our analysis and that by NICE, since we based our analysis on diagnostic accuracies compared with ICA-FFR, rather than diagnostic accuracies of an anatomical assessment of ICA alone, which is a well-recognised limitation acknowledged in the NICE economic assessment. However, in our analysis, CTCA features as part of a cost-effective strategy following the results of a positive functional test, at low CET values per correct diagnosis.

There has been much debate on the use of direct first line invasive coronary angiography, and it has been shown that using ICA alone has a low diagnostic yield for the detection of CAD.^8^ A large multicentre study demonstrated that patients that underwent ICA-FFR guided management (functional) had better clinical outcomes compared with that based on visual estimation of stenosis (anatomical).^4^ Our analysis indicates that direct ICA is a dominated strategy (less effective and more expensive) compared with sequential testing using functional testing as the first line. Thus, our study supports the notion that ICA is not an ideal strategy for detection of functional CAD, from a cost-effectiveness perspective.

We found that CMR is a cost-effective first-line strategy across a wide range of CET values, particularly in patients with low risk of CAD. Ultimately, the cost-effectiveness of diagnostic tests depends on the benefits to patients that treating the underlying disease provides. In order to appropriately quantify these potential benefits, analysis of the downstream costs and outcomes of disease management is required. Nonetheless, we can make a comparison with acceptable threshold values for a year in full health. NICE applies a threshold of £20 000–£30 000 per quality-adjusted life-year (QALY, equivalent to a year in full health) when considering evidence on cost-effectiveness.^18^ If a correct diagnosis is believed to lead to a health gain of more than 0.1 QALYs but less than 0.5 QALYs the implied CET for a correct diagnosis would range from £2000 to £15 000, with a higher health gain implying a higher threshold. This range broadly equates with the range of CET values of £3000–£17 000 over which CMR followed by CTCA and the ICA-FFR is cost-effective at a pretest probability of 37%. Hence CMR +CTCA+ICA-FFR is likely to be cost-effective in the UK if the health gain from a correct diagnosis lies between 0.1 and 0.5 QALYs.

Several studies have evaluated the cost-effectiveness of diagnostic strategies for the detection of CAD in patients with stable angina. There is a wide heterogeneity in the methodologies applied and results obtained, which include short-term and long-term model-based economic evaluations, as well as trial-based cost-effectiveness analysis.^19^ Initial studies that compared CTCA and CMR using a model-based cost-effectiveness analysis demonstrated that CTCA was more cost-effective than CMR for the diagnosis of CAD.^20^ However, in contrast to the current study, ICA alone was used as the reference standard rather than ICA-FFR, which would otherwise favour an anatomical test such as CTCA.

In contrast to our findings, the Evaluation of Integrated Cardiac Imaging in Ischemic Heart Disease (EVINCI) study suggested that combined testing with anatomical and functional imaging was a cost-effective strategy.^21^ However, the EVINCI study was also based on a diagnostic end point of obstructive CAD defined by >50% stenosis on ICA rather than ICA-FFR.

Studies that have compared SPECT to CMR using ICA as the reference standard, concluded that CMR was more cost effective compared with SPECT in the UK^22^ and in Germany.^23^ In a recent economic analysis using data from the CE-MARC 2 trial, CMR and SPECT were compared against NICE recommendations in guiding management of CAD, CMR was found to be the most cost-effective strategy regardless of pretest probability.^24^ In another study, in which long-term modelling with a range of tests was examined, CTCA followed by stress testing and invasive angiography was the most cost-effective strategy,^25^ and the findings are different to our current study, which may in part relate to the more contemporary data on diagnostic accuracies used in this current study. More recently, efforts have been made to determine the cost-effectiveness of diagnostics using ICA-FFR as the reference test. A model-based economic evaluation which compared CTCA, CMR, SPECT and ICA found that CMR is cost-effective at a pretest likelihood of 32%.^26^

To the authors’ knowledge, our study is the first economic evaluation which has incorporated all the available diagnostic tests for functional CAD over a broad range of pretest probabilities using ICA-FFR as the reference standard. We believe that the inclusion of all reasonable alternative strategies is important to provide unbiased results to guide decision making. These findings may be used as an adjunct to the ESC chronic coronary syndrome guideline (based primarily on clinical effectiveness) and provide complementary information for decision making.

The interpretation of our findings needs to be considered with the limitations intrinsic to the analysis. First, our analysis applied a short time horizon in which only immediate decisions were captured. We did not include the downstream costs of potential future tests, treatment of stable CAD and costs of treating cardiovascular events. We deliberately chose not to pursue the long-term modelling as there is considerable debate as to the long term outcomes and downstream clinical management of patients with stable CAD. For instance, given the recent findings of the ISCHEMIA trial,^27^ it may be that a correct diagnosis followed by medical therapy alone is all that is needed for patients with stable CAD. Future work is warranted to compare strategies which include the long-term costs and benefits to patients when long term outcome data are available. This would improve decision making by allowing a fuller consideration of the health impact of correct diagnosis. Second, in this decision modelling process, the payer perspective was that of the UK NHS healthcare system, and therefore the extrapolation of findings needs consideration of local costs in alternative healthcare settings. Third, the model assumes that all patients were suitable for all tests. However, in clinical practice, certain tests may not be suitable for all patients. For instance, CMR may be contraindicated for patients with metallic implants, SE may result in poor image quality in patients with large body habitus and CTCA may not be suitable for patients with allergy to iodinated contrast agents. However, we have provided illustrative ICERs for all the available options that may be considered by each local health economy.

Finally, the model considers patients with new onset stable angina, and not patients with previous established CAD with previous stents or coronary artery bypass grafting.

## Conclusions

CMR is a cost-effective first line test for functional CAD across a range of threshold values placed on a correct diagnosis. SPECT may be cost-effective at a very low CET. ICA is not an appropriate first-line strategy, although direct ICA-FFR may be considered in patients with a high pretest probability of CAD. These findings support the use of non-invasive testing for diagnosis of CAD and serve to inform clinical decision making on the use of these tests from a cost-effectiveness perspective.

## Data Availability

All data relevant to the study are included in the article or uploaded as online supplemental information.

## References

[1] Cardiovascular diseases (CVDs). Available: http://www.who.int/mediacentre/factsheets/fs317/en/

[2] NICE. Chest pain of recent onset. Assessment and diagnosis of recent onset chest pain or discomfort of suspected cardiac origin (update). In: NICE guideline CG95. National Institute for health and care excellence, 2016.

[3] Knuuti J, Wijns W, Saraste A, et al. 2019 ESC guidelines for the diagnosis and management of chronic coronary syndromes. Eur Heart J 2020;41:407–77. 10.1093/eurheartj/ehz425 31504439

[4] Tonino PAL, De Bruyne B, Pijls NHJ, et al. Fractional flow reserve versus angiography for guiding percutaneous coronary intervention. N Engl J Med 2009;360:213–24. 10.1056/NEJMoa0807611 19144937

[5] De Bruyne B, Pijls NHJ, Kalesan B, et al. Fractional flow reserve-guided PCI versus medical therapy in stable coronary disease. N Engl J Med 2012;367:991–1001. 10.1056/NEJMoa1205361 22924638

[6] Knuuti J, Ballo H, Juarez-Orozco LE, et al. The performance of non-invasive tests to rule-in and rule-out significant coronary artery stenosis in patients with stable angina: a meta-analysis focused on post-test disease probability. Eur Heart J 2018;39:3322–30. 10.1093/eurheartj/ehy267 29850808

[7] Danad I, Szymonifka J, Twisk JWR. Diagnostic performance of cardiac imaging methods to diagnose ischaemia-causing coronary artery disease when directly compared with fractional flow reserve as a reference standard: a meta-analysis. Eur Heart J 2016;38:991–8. 10.1093/eurheartj/ehw095 PMC5381594 27141095

[8] Patel MR, Peterson ED, Dai D, et al. Low diagnostic yield of elective coronary angiography. N Engl J Med 2010;362:886–95. 10.1056/NEJMoa0907272 20220183 PMC3920593

[9] Knuuti J, Wijns W, Saraste A. 2019 ESC guidelines for the diagnosis and management of chronic coronary syndromes. Eur Heart J 2019;41. 10.1093/eurheartj/ehz425 31504439

[10] Noto TJ, Johnson LW, Krone R, et al. Cardiac catheterization 1990: a report of the Registry of the Society for Cardiac Angiography and Interventions (SCA&I). Cathet Cardiovasc Diagn 1991;24:75–83. 10.1002/ccd.1810240202 1742788

[11] Bruder O, Wagner A, Lombardi M, et al. European Cardiovascular Magnetic Resonance (EuroCMR) registry--multi national results from 57 centers in 15 countries. J Cardiovasc Magn Reson 2013;15:9. 10.1186/1532-429X-15-9 23331632 PMC3564740

[12] Varga A, Garcia MAR, Picano E, et al. Safety of stress echocardiography (from the International stress echo complication registry). Am J Cardiol 2006;98:541–3. 10.1016/j.amjcard.2006.02.064 16893714

[13] Cerqueira MD, Verani MS, Schwaiger M, et al. Safety profile of adenosine stress perfusion imaging: results from the Adenoscan multicenter trial registry. J Am Coll Cardiol 1994;23:384–9. 10.1016/0735-1097(94)90424-3 8294691

[14] Lu MT, Douglas PS, Udelson JE, et al. Safety of coronary CT angiography and functional testing for stable chest pain in the promise trial: a randomized comparison of test complications, incidental findings, and radiation dose. J Cardiovasc Comput Tomogr 2017;11:373–82. 10.1016/j.jcct.2017.08.005 28838846 PMC6201309

[15] NHS Improvement 2018. Reference Cost Collection: National Schedule of Reference Costs - Year 2017-18 - NHS trust and NHS foundation trusts. Available: https://improvement.nhs.uk/resources/reference-costs/ [Accessed 10th November 2019].

[16] Drummond MF, Sculpher MJ, Claxton K, et al. Methods for the economic evaluation of health care programmes. Oxford University Press: Oxford: Oxford, 2015.

[17] van Hout BA, Al MJ, Gordon GS, et al. Costs, effects and C/E-ratios alongside a clinical trial. Health Econ 1994;3:309–19. 10.1002/hec.4730030505 7827647

[18] NICE. Guide to the methods of technology appraisal 2013, 2013. Available: https://www.nice.org.uk/process/pmg9/resources/guide-to-the-methods-of-technology-appraisal-2013-pdf-2007975843781 27905712

[19] van Waardhuizen CN, Khanji MY, Genders TSS, et al. Comparative cost-effectiveness of non-invasive imaging tests in patients presenting with chronic stable chest pain with suspected coronary artery disease: a systematic review. Eur Heart J Qual Care Clin Outcomes 2016;2:245–60. 10.1093/ehjqcco/qcw029 29474724

[20] Dewey M, Hamm B. Cost effectiveness of coronary angiography and calcium scoring using CT and stress MRI for diagnosis of coronary artery disease. Eur Radiol 2007;17:1301–9. 10.1007/s00330-006-0439-3 17031453

[21] Lorenzoni V, Bellelli S, Caselli C, et al. Cost-Effectiveness analysis of stand-alone or combined non-invasive imaging tests for the diagnosis of stable coronary artery disease: results from the EVINCI study. Eur J Health Econ 2019;20:1437–49. 10.1007/s10198-019-01096-5 31410670 PMC6856023

[22] Walker S, Girardin F, McKenna C, et al. Cost-Effectiveness of cardiovascular magnetic resonance in the diagnosis of coronary heart disease: an economic evaluation using data from the CE-MARC study. Heart 2013;99:873–81. 10.1136/heartjnl-2013-303624 23591668

[23] Boldt J, Leber AW, Bonaventura K, et al. Cost-Effectiveness of cardiovascular magnetic resonance and single-photon emission computed tomography for diagnosis of coronary artery disease in Germany. J Cardiovasc Magn Reson 2013;15:30. 10.1186/1532-429X-15-30 23574690 PMC3688498

[24] Walker S, Cox E, Rothwell B, et al. Cost-Effectiveness of cardiovascular imaging for stable coronary heart disease. Heart 2021;107:381-388. 10.1136/heartjnl-2020-316990 32817271 PMC7892375

[25] Genders TSS, Petersen SE, Pugliese F, et al. The optimal imaging strategy for patients with stable chest pain: a cost-effectiveness analysis. Ann Intern Med 2015;162:474–84. 10.7326/M14-0027 25844996

[26] Ge Y, Pandya A, Steel K, et al. Cost-Effectiveness analysis of stress cardiovascular magnetic resonance imaging for stable chest pain syndromes. JACC Cardiovasc Imaging 2020;13:1505–17. 10.1016/j.jcmg.2020.02.029 32417337

[27] Maron DJ, Hochman JS, Reynolds HR, et al. Initial invasive or conservative strategy for stable coronary disease. N Engl J Med 2020;382:1395–407. 10.1056/NEJMoa1915922 32227755 PMC7263833

