# Supplementary material for: Cost-effectiveness in diagnosis of stable angina patients: a decision-analytical modelling approach
Source: Open Heart. 2022 Apr 4;9(1):e001700. doi: 10.1136/openhrt-2021-001700 (PMC8981340; doi:10.1136/openhrt-2021-001700)
Supplement: Supplementary data [file openhrt-2021-001700supp001.pdf]

Supplementary data

Table A1 details all of the possible strategies that were considered, the ICERs and which ones were dominated or extendedly dominated.

Table A2 details the parameters that were used to derive the cost effectiveness model.

Table A3 details the results for all strategies, the ICER and whether the strategy was dominated or extendedly dominated.

Table A4 presents the breakdown of the scenario analysis according to the pretest probability of CAD.

Figure A4 details the scenario analyses of pre-test likelihood and cost effectiveness threshold per correct diagnosis.

Tables

Strategy	First test	Second test	Third test
0	No testing		
1	ICA-FFR		
**Standalone testing which triggers ICA-FFR with a positive test result**			
2	CTCA	ICA-FFR	
3	CMR	ICA-FFR	
4	PET	ICA-FFR	
5	SE	ICA-FFR	
6	ICA	ICA-FFR	
7	SPECT	ICA-FFR	
**Anatomical imaging first with CT followed by functional imaging and ICA-FFR**			
8	CTCA	CMR	ICA-FFR
9	CTCA	SE	ICA-FFR
10	CTCA	SPECT	ICA-FFR
11	CTCA	PET	ICA-FFR
12	CTCA	CT-FFR	ICA-FFR
13*	CTCA	ICA	ICA-FFR
**Functional imaging first followed by anatomical CT and then ICA-FFR**			
14	SE	CTCA	ICA-FFR
15	SPECT	CTCA	ICA-FFR
16	CMR	CTCA	ICA-FFR
17	PET	CTCA	ICA-FFR

Table A1. 17 diagnostic strategies used in the model. CTCA: Computed Tomography Coronary Angiography. CT-FFR: Computed Tomography derived Fractional Flow Reserve. CMR: Cardiovascular Magnetic Resonance. ICA: Invasive Coronary Angiography. ICA-FFR: Invasive Coronary Angiography and Fractional Flow Reserve. Positron Emission Tomography. SE: Stress Echocardiography. SPECT: Single-Photon Emission Computed Tomography.

Test	Parameter	Mean (SE)	Distribution	Source
ICA-FFR	Cost (£)	2,153 (430.60)	Gamma	2017/2018 NHS Reference Costs
	Complication cost (£)	1,435.30 (353.82)	Gamma	Weighted average of 2017/2018 NHS Reference cost of the following HRG codes: EB07, EB03, EB10, EB14, AA29, AA22, YQ50 and WH05Z
	Sensitivity	100%	-	Assumption
	Specificity	100%	-	Assumption
	Risk of death	0.072% (0.0001)	Beta	Noto et al (1991)
	Risk of non-fatal complication	0.94% (0.002)	Beta	
CTCA	Cost (£)	262 (52.40)	Gamma	2017/2018 NHS Reference Costs
	Complication cost (£)	563.04 (112.61)	Gamma	Weighted average of 2017/2018 NHS Reference cost of the following HRG codes: WH05 and EB03
	Sensitivity	93% (0.016)	Beta	Knuuti et al (2018)
	Specificity	53% (0.127)	Beta	
	Risk of death	0%	-	Lu et al (2017)
	Risk of non-fatal complication	0.80% (0.0013)	Beta	
CT-FFR	Cost (£)	962 (192.40)	Gamma	2017/2018 NHS Reference Costs

Test	Parameter	Mean (SE)	Distribution	Source
	Sensitivity	90% (0.016)	Beta	Danad et al (2017)
	Specificity	71% (0.0280)	Beta	Danad et al (2017)
	Risk of death	0%	-	Assumed to be 0 to avoid double counting
	Risk of non-fatal complication	0%	-	Assumed to be 0 to avoid double counting
CMR	Cost (£)	389 (77.80)	Gamma	2017/2018 NHS Reference Costs
	Complication cost (£)	1,253 (250.60)	Gamma	Weighted average of 2017/2018 NHS Reference cost of the following HRG codes: EB07, EB03, EB10, WH05Z
	Sensitivity	89% (0.017)	Beta	Knuuti et al (2018)
	Specificity	87% (0.023)	Beta	Knuuti et al (2018)
	Risk of death	0%	-	Bruder et al (2013)
	Risk of non-fatal complication	0.10% (0.0003)	Beta	Bruder et al (2013)
	Cost (£)	498 (99.60)	Gamma	2017/2018 NHS Reference Costs
	Complication cost (£)	1962.83 (392.57)	Gamma	Assumed to be equal to SPECT
PET	Sensitivity	89% (0.0224)	Beta	Knuuti et al (2018)
	Specificity	85% (0.0177)	Beta	Knuuti et al (2018)
	Risk of death	0%	-	Assumed to be equal to SPECT

Test	Parameter	Mean (SE)	Distribution	Source
	Risk of non-fatal complication	0.10% (0.0003)	Beta	Assumed to be equal to SPECT
SE	Cost (£)	250 (50)	Gamma	2017/2018 NHS Reference Costs
	Complication cost (£)	1,192.06 (238.41)	Gamma	Weighted average of 2017/2018 NHS Reference cost of the following HRG codes: EB07, EB03, EB10, EB14, AA35 and AA22
	Sensitivity	77% (0.05)	Beta	Danad et al (2017)
	Specificity	75% (0.087)	Beta	Danad et al (2017)
	Risk of death	0.00069% (0.00002)	Beta	Varga et al (2006)
	Risk of non-fatal complication	0.10% (0.0001)	Beta	Varga et al (2006)
ICA	Cost (£)	1,726 (345.20)	Gamma	2017/2018 NHS Reference Costs
	Complication cost (£)	1349.81 (269.96)	Gamma	Weighted average of 2017/2018 NHS Reference cost of the following HRG codes: EB07, EB03, EB10, EB14, AA29, AA22, YQ50 and WH05Z
	Sensitivity	68% (0.05)	Beta	Knuuti et al (2018)
	Specificity	73% (0.836)	Beta	Knuuti et al (2018)
	Risk of death	0.072% (0.0001)	Beta	Noto et al (1991)
	Risk of non-fatal complication	0.74% (0.0015)	Beta	Noto et al (1991)

Test	Parameter	Mean (SE)	Distribution	Source
SPECT	Cost (£)	272 (54.4)	Gamma	2017/2018 NHS Reference Costs
	Complication cost (£)	1,962.83 (490.71)	Gamma	Weighted average of 2017/2018 NHS Reference cost of the following HRG codes: EB10, DZ19, and DZ20
	Sensitivity	73% (0.0593)	Beta	Knuuti et al (2018)
	Specificity	83% (0.0413)	Beta	Knuuti et al (2018)
	Risk of death	0%	-	Cerqueira et al (1994)
	Risk of non-fatal complication	0.10% (0.0003)	Beta	Cerqueira et al (1994)

Table A2. Model parameters. CTCA: Computed Tomography Coronary Angiography. CT-FFR: Computed Tomography derived Fractional Flow Reserve. CMR: Cardiovascular Magnetic Resonance. ICA: Invasive Coronary Angiography. ICA-FFR: Invasive Coronary Angiography and Fractional Flow Reserve. PET: Positron Emission Tomography. SE: Stress Echocardiography. SPECT: Single-Photon Emission Computed Tomography. NHS: National Health Service

Table A3. Outline all of the strategies that were evaluated. The options that were dominated or extendedly dominated are identified.

Strategy no.	Description	Expected deaths	Expected complications	Expected proportion of incorrect diagnoses	Expected proportion of correct diagnoses	Expected cost	Dominance
1	ICA-FFR	0.08%	0.94%	0.00%	98.97%	£2,176.02	Non-dominated
2	CTCA + ICA-FFR	0.05%	1.41%	2.63%	95.91%	£1,656.47	Dominated
3	CMR + ICA-FFR	0.03%	0.46%	4.16%	95.35%	£1,291.80	Non-dominated
4	PET + ICA-FFR	0.04%	0.50%	4.11%	95.35%	£1,431.49	Dominated
5	SE + ICA-FFR	0.04%	0.52%	8.71%	90.73%	£1,221.70	Dominated
6	ICA + FFR	0.12%	1.24%	11.84%	86.80%	£2,473.56	Dominated
7	SPECT +ICA-FFR	0.03%	0.46%	10.16%	89.35%	£1,103.96	Dominated
8	CTCA + CMR + ICA-FFR	0.02%	1.10%	5.25%	93.59%	£1,090.84	Dominated
9	CTCA + SE + ICA-FFR	0.02%	1.14%	8.20%	90.64%	£1,045.88	Extendedly dominated
10	CTCA + SPECT + ICA-FFR	0.02%	1.10%	9.12%	89.76%	£970.47	Extendedly dominated
11	CTCA + PET + ICA-FFR	0.02%	1.12%	5.26%	93.60%	£1,180.00	Dominated
12	CTCA + FFRCT + ICA-FFR	0.03%	1.09%	4.32%	94.55%	£2,202.17	Dominated
13	CTCA + ICA + FFR	0.08%	1.60%	10.20%	88.13%	£1,959.60	Dominated
14	SE + CTCA + ICA-FFR	0.02%	0.73%	9.88%	89.37%	£990.23	Dominated
15	SPECT + CTCA + ICA-FFR	0.02%	0.63%	11.16%	88.18%	£905.90	Non-dominated
16	CMR + CTCA + ICA-FFR	0.02%	0.65%	5.25%	94.08%	£1,075.93	Non-dominated
17	PET + CTCA +ICA-FFR	0.02%	0.70%	5.24%	94.04%	£1,209.28	Dominated

Table A4. Scenario analysis on pre-test probability

Pre-test probability	Strategy no.	Description	Expected costs	Expected proportion of correct diagnoses	Expected proportion of complications	Expected proportion of deaths	NMB	Probability of being the most cost-effective strategy
**CET value of £2,000 per correct diagnosis**								
15%	15	SPECT + CTCA + ICA-FFR	£633.04	95.22%	0.43%	0.01%	£1,271.34	47%
25.00%	15	SPECT + CTCA + ICA-FFR	£742.42	92.25%	0.52%	0.01%	£1,102.53	47%
35.00%	15	SPECT + CTCA + ICA-FFR	£863.64	88.96%	0.60%	0.02%	£915.64	44%
45.00%	15	SPECT + CTCA + ICA-FFR	£995.88	85.77%	0.70%	0.02%	£719.46	42%
55.00%	15	SPECT + CTCA + ICA-FFR	£1,142.66	82.70%	0.81%	0.03%	£511.44	41%
65.00%	15	SPECT + CTCA + ICA-FFR	£1,293.45	78.97%	0.91%	0.03%	£286.02	33%
75.00%	15	SPECT + CTCA + ICA-FFR	£1,469.07	75.79%	1.02%	0.04%	£46.66	29%
85.00%	7	SPECT +ICA-FFR	£1,666.85	76.22%	0.70%	0.05%	-£142.47	26%
**CET value of £3,000 per correct diagnosis**								
15%	15	SPECT + CTCA + ICA-FFR	£637.88	95.25%	0.43%	0.01%	£2,219.55	40%
25.00%	15	SPECT + CTCA + ICA-FFR	£746.65	92.19%	0.51%	0.02%	£2,019.19	34%
35.00%	15	SPECT + CTCA + ICA-FFR	£868.55	89.17%	0.61%	0.02%	£1,806.42	31%
45.00%	16	CMR + CTCA + ICA-FFR	£1,193.56	92.76%	0.74%	0.03%	£1,589.34	32%
55.00%	16	CMR + CTCA + ICA-FFR	£1,378.19	90.93%	0.88%	0.03%	£1,349.71	26%
65.00%	16	CMR + CTCA + ICA-FFR	£1,573.38	88.98%	1.01%	0.04%	£1,096.03	19%
75.00%	3	CMR + ICA-FFR	£1,914.89	91.00%	0.73%	0.06%	£815.22	12%
85.00%	1	ICA-FFR	£2,166.69	98.97%	0.94%	0.08%	£802.51	80%

Pre-test probability	Strategy no.	Description	Expected costs	Expected proportion of correct diagnoses	Expected proportion of complications	Expected proportion of deaths	NMB	Probability of being the most cost-effective strategy
**CET value of £5,000 per correct diagnosis**								
15%	16	CMR + CTCA + ICA-FFR	£745.54	97.70%	0.39%	0.01%	£4,139.60	38%
25.00%	16	CMR + CTCA + ICA-FFR	£877.13	96.19%	0.50%	0.02%	£3,932.29	47%
35.00%	16	CMR + CTCA + ICA-FFR	£1,023.78	94.59%	0.61%	0.02%	£3,705.78	49%
45.00%	16	CMR + CTCA + ICA-FFR	£1,201.97	92.82%	0.75%	0.03%	£3,438.85	47%
55.00%	16	CMR + CTCA + ICA-FFR	£1,384.03	90.97%	0.88%	0.03%	£3,164.40	34%
65.00%	3	CMR + ICA-FFR	£1,739.05	92.13%	0.65%	0.05%	£2,867.28	24%
75.00%	1	ICA-FFR	£2,178.37	98.97%	0.94%	0.08%	£2,770.38	73%
85.00%	1	ICA-FFR	£2,175.19	98.97%	0.94%	0.08%	£2,773.40	99%
**CET value of £7,500 per correct diagnosis**								
15%	16	CMR + CTCA + ICA-FFR	£738.62	97.70%	0.39%	0.01%	£6,589.16	50%
25.00%	16	CMR + CTCA + ICA-FFR	£880.02	96.18%	0.50%	0.02%	£6,333.37	57%
35.00%	16	CMR + CTCA + ICA-FFR	£1,039.49	94.59%	0.61%	0.02%	£6,055.12	53%
45.00%	16	CMR + CTCA + ICA-FFR	£1,205.47	92.80%	0.75%	0.03%	£5,754.55	44%
55.00%	16	CMR + CTCA + ICA-FFR	£1,388.79	90.98%	0.88%	0.03%	£5,435.00	24%
65.00%	1	ICA-FFR	£2,169.79	98.97%	0.94%	0.08%	£5,253.31	57%
75.00%	1	ICA-FFR	£2,180.12	98.97%	0.94%	0.08%	£5,242.90	95%
85.00%	1	ICA-FFR	£2,167.80	98.97%	0.94%	0.08%	£5,255.18	100%
**CET value of £10,000 per correct diagnosis**								
15%	16	CMR + CTCA + ICA-FFR	£737.57	97.70%	0.39%	0.01%	£9,032.81	60%

Pre-test probability	Strategy no.	Description	Expected costs	Expected proportion of correct diagnoses	Expected proportion of complications	Expected proportion of deaths	NMB	Probability of being the most cost-effective strategy
25.00%	16	CMR + CTCA + ICA-FFR	£876.13	96.17%	0.50%	0.02%	£8,741.16	62%
35.00%	16	CMR + CTCA + ICA-FFR	£1,030.93	94.57%	0.62%	0.02%	£8,425.97	52%
45.00%	16	CMR + CTCA + ICA-FFR	£1,201.95	92.78%	0.74%	0.03%	£8,075.82	29%
55.00%	3	CMR + ICA-FFR	£1,572.96	93.33%	0.58%	0.05%	£7,759.85	24%
65.00%	1	ICA-FFR	£2,159.87	98.97%	0.94%	0.08%	£7,737.33	84%
75.00%	1	ICA-FFR	£2,156.34	98.97%	0.94%	0.08%	£7,741.02	100%
85.00%	1	ICA-FFR	£2,168.35	98.97%	0.94%	0.08%	£7,729.09	100%
**CET value of £15,000 per correct diagnosis**								
15%	16	CMR + CTCA + ICA-FFR	£735.71	97.69%	0.39%	0.01%	£13,918.48	67%
25.00%	16	CMR + CTCA + ICA-FFR	£888.94	96.18%	0.50%	0.02%	£13,538.14	56%
35.00%	16	CMR + CTCA + ICA-FFR	£1,028.96	94.55%	0.62%	0.02%	£13,153.49	38%
45.00%	3	CMR + ICA-FFR	£1,410.39	94.48%	0.51%	0.04%	£12,762.16	27%
55.00%	1	ICA-FFR	£2,158.18	98.97%	0.94%	0.08%	£12,687.71	79%
65.00%	1	ICA-FFR	£2,162.09	98.97%	0.94%	0.08%	£12,683.86	98%
75.00%	1	ICA-FFR	£2,164.70	98.97%	0.94%	0.08%	£12,681.19	100%
85.00%	1	ICA-FFR	£2,170.22	98.97%	0.94%	0.08%	£12,675.73	100%
**CET value of £20,000 per correct diagnosis**								
15%	16	CMR + CTCA + ICA-FFR	£737.60	97.69%	0.38%	0.01%	£18,801.05	66%
25.00%	16	CMR + CTCA + ICA-FFR	£881.40	96.20%	0.50%	0.02%	£18,359.30	50%
35.00%	3	CMR + ICA-FFR	£1,248.38	95.66%	0.44%	0.03%	£17,884.35	31%
45.00%	1	ICA-FFR	£2,158.89	98.97%	0.94%	0.08%	£17,635.79	60%

Pre-test probability	Strategy no.	Description	Expected costs	Expected proportion of correct diagnoses	Expected proportion of complications	Expected proportion of deaths	NMB	Probability of being the most cost-effective strategy
55.00%	1	ICA-FFR	£2,184.68	98.97%	0.94%	0.08%	£17,609.95	95%
65.00%	1	ICA-FFR	£2,177.10	98.97%	0.94%	0.08%	£17,617.81	100%
75.00%	1	ICA-FFR	£2,174.90	98.97%	0.94%	0.08%	£17,619.73	100%
85.00%	1	ICA-FFR	£2,164.07	98.97%	0.94%	0.08%	£17,630.56	100%
**CET value of £30,000 per correct diagnosis**								
15%	16	CMR + CTCA + ICA-FFR	£731.84	97.72%	0.39%	0.01%	£28,584.48	63%
25.00%	16	CMR + CTCA + ICA-FFR	£876.80	96.18%	0.50%	0.02%	£27,976.81	30%
35.00%	1	ICA-FFR	£2,166.30	98.97%	0.94%	0.08%	£27,525.48	49%
45.00%	1	ICA-FFR	£2,180.69	98.97%	0.94%	0.08%	£27,511.04	93%
55.00%	1	ICA-FFR	£2,139.13	98.97%	0.94%	0.08%	£27,552.69	100%
65.00%	1	ICA-FFR	£2,174.04	98.97%	0.94%	0.08%	£27,517.77	100%
75.00%	1	ICA-FFR	£2,165.04	98.97%	0.94%	0.08%	£27,527.26	100%
85.00%	1	ICA-FFR	£2,172.31	98.97%	0.94%	0.08%	£27,519.61	100%
**CET value of £50,000 per correct diagnosis**								
15%	16	CMR + CTCA + ICA-FFR	£738.32	97.70%	0.39%	0.01%	£48,110.28	37%
25.00%	3	CMR + ICA-FFR	£1,083.60	96.87%	0.37%	0.03%	£47,351.17	34%
35.00%	1	ICA-FFR	£2,155.01	98.97%	0.94%	0.08%	£47,331.81	95%
45.00%	1	ICA-FFR	£2,170.51	98.97%	0.94%	0.08%	£47,316.71	100%
55.00%	1	ICA-FFR	£2,177.94	98.97%	0.94%	0.08%	£47,308.70	100%
65.00%	1	ICA-FFR	£2,151.75	98.97%	0.94%	0.08%	£47,334.97	100%
75.00%	1	ICA-FFR	£2,188.27	98.97%	0.94%	0.08%	£47,298.28	100%

Pre-test probability	Strategy no.	Description	Expected costs	Expected proportion of correct diagnoses	Expected proportion of complications	Expected proportion of deaths	NMB	Probability of being the most cost-effective strategy
85.00%	1	ICA-FFR	£2,172.43	98.97%	0.94%	0.08%	£47,313.73	100%
